# Alpha-Lipoic Acid and Benfotiamine in Diabetic Peripheral Neuropathy: A Critical Review of Mechanistic Rationale and Clinical Evidence Within a Nutritional Therapeutic Framework

**DOI:** 10.3390/nu18101538

**Published:** 2026-05-12

**Authors:** Alin Ciubotaru, Cristina Grosu, Daniel Alexa, Laura-Elena Cucu, Thomas Gabriel Schreiner, Cătălina Elena Bistriceanu, Alexandra Maştaleru, Doina Azoicāi, Albert Vamanu, Alexandru Patrascu, Dan Iulian Cuciureanu, Emilian Bogdan Ignat

**Affiliations:** 1 Grigore T. Popa University of Medicine and Pharmacy, 700115 Iasi, Romania; alinciubotaru94@yahoo.com (A.C.); daniel.alexa@umfiasi.ro (D.A.); catalina-elena.bistriceanu@umfiasi.ro (C.E.B.); alexandra.mastaleru@umfiasi.ro (A.M.); doina.azoicai@gmail.com (D.A.); cuciureanudan@yahoo.com (D.I.C.);; 2 Basic and Clinical Neuroscience Department, Institute of Psychiatry, Psychology and Neuroscience, King’s College London, London SE5 8AF, UK; albert.vamanu@kcl.ac.uk; 3 Apollonia University, 700511 Iasi, Romania; patrascu_alex@yahoo.com

**Keywords:** diabetic peripheral neuropathy, alpha-lipoic acid, benfotiamine, metabolic therapy, oxidative stress, advanced glycation end products, transketolase, nutritional intervention

## Abstract

Background: Diabetic peripheral neuropathy (DPN) affects up to 50% of diabetes patients and is driven by hyperglycemia-induced oxidative stress, mitochondrial dysfunction, polyol pathway activation, advanced glycation end-product formation, and inflammation. Current management is largely symptomatic, prompting interest in metabolic/nutritional therapies. This review critically evaluates the mechanistic rationale and clinical evidence for alpha-lipoic acid (ALA) and benfotiamine as adjunctive treatments for DPN. Methods: A structured narrative review of PubMed/MEDLINE was conducted using predefined keywords for DPN, oxidative stress, metabolic therapy, and thiamine derivatives. Randomized controlled trials, clinical studies, systematic reviews, and relevant experimental studies were included. Evidence was synthesized qualitatively with emphasis on mechanistic plausibility, clinical efficacy, intervention duration, and methodological rigor. Results: ALA consistently improves short-term symptoms across multiple randomized trials. The long-term NATHAN 1 trial reported a marginal, borderline significant effect on the primary composite endpoint (NIS-LL, *p* = 0.05) without significant improvements in nerve conduction studies; therefore, evidence for functional stabilization is very limited and inconclusive. ALA’s effects are attributed to antioxidant activity, mitochondrial protection, and improved microvascular function. Benfotiamine has a strong biochemical rationale (transketolase activation, diversion of glycolytic intermediates from damaging pathways), but clinical evidence remains limited to short-duration, symptom-based studies, with no large-scale, long-term trials published. Conclusions: Both agents target key pathways in DPN pathogenesis. ALA is the most established adjunctive metabolic therapy for symptomatic DPN, although no study has demonstrated structural nerve regeneration or a definitive disease-modifying effect. Benfotiamine is biologically plausible but requires further validation in long-term randomized trials with structural and biomarker-based endpoints. Outside of documented thiamine deficiency, its routine use cannot be recommended based on current evidence.

## 1. Introduction

Diabetic peripheral neuropathy (DPN) represents the most prevalent chronic complication of diabetes mellitus, affecting approximately 30–50% of individuals over their lifetime [1,2]. It is characterized by a length-dependent, distal symmetric polyneuropathy that primarily involves small sensory fibers in its early stages, followed by progressive large-fiber and, eventually, motor involvement. Clinically, DPN manifests with numbness, paresthesia, dysesthesia, neuropathic pain, and impaired vibration perception, ultimately predisposing patients to foot ulceration and lower-extremity amputation [2]. Globally, DPN constitutes the leading cause of neuropathic pain and accounts for a substantial proportion of non-traumatic amputations [3].

Although chronic hyperglycemia remains the principal etiological factor, the pathogenesis of DPN is multifactorial. Intensive glycemic control has been shown to reduce neuropathy incidence in type 1 diabetes, as demonstrated in the DCCT/EDIC cohort [4]. However, its protective effect in type 2 diabetes is less pronounced, and neuropathy progression frequently persists despite optimized metabolic control [2,5]. This observation underscores the contribution of additional metabolic and vascular mechanisms beyond mere glucose toxicity.

At the molecular level, chronic hyperglycemia induces mitochondrial overproduction of reactive oxygen species (ROS), which has been proposed as a unifying mechanism underlying diabetic microvascular complications [3]. This oxidative stress triggers several downstream pathways, including activation of the polyol pathway, increased formation of advanced glycation end products (AGEs), protein kinase C (PKC) activation, and increased hexosamine pathway flux [3,6]. These interrelated processes converge to impair neuronal energy homeostasis, axonal transport, microvascular perfusion, and neurotrophic support, ultimately leading to axonal degeneration and distal fiber loss [7].

Current management of DPN is largely symptomatic. First-line pharmacological agents such as duloxetine, pregabalin, and gabapentin provide partial analgesic benefit but do not address the underlying metabolic injury cascades that drive progressive nerve damage [2]. Consequently, there is growing interest in pathogenesis-oriented strategies aimed at modulating oxidative stress, mitochondrial dysfunction, and aberrant glucose-derived metabolic flux.

Within this context, nutritionally derived metabolic modulators have attracted particular attention. Alpha-lipoic acid (ALA), an endogenous mitochondrial cofactor with potent antioxidant properties, has been extensively investigated for its ability to reduce oxidative stress and improve microvascular function [4,5,6,7]. Benfotiamine, a lipid-soluble derivative of thiamine (vitamin B1), enhances transketolase activity and may redirect glycolytic intermediates away from pathways implicated in diabetic complications [8,9].

ALA and benfotiamine were selected for this review because they represent two distinct, mechanistically complementary approaches to metabolic intervention—one targeting downstream oxidative stress (ALA) and the other targeting upstream metabolic flux diversion via transketolase activation (benfotiamine). Both have reached clinical use as over-the-counter supplements, creating an urgent need for critical evidence synthesis, unlike other nutritional agents (e.g., acetyl-L-carnitine and vitamin D), which have either less mechanistic rationale or weaker clinical uptake in DPN.

In this review, the term ‘nutritional’ refers to interventions derived from naturally occurring compounds (alpha-lipoic acid, a mitochondrial cofactor, and benfotiamine, a synthetic thiamine derivative) that are used as dietary supplements rather than as registered pharmaceuticals. This is distinct from whole-diet approaches or essential nutrient deficiency correction. The ‘nutritional therapeutic framework’ therefore denotes the use of supplement-level metabolic modulators within a broader strategy that includes glycemic control and symptomatic pharmacotherapy, not a replacement for conventional diabetes nutrition therapy.

Despite decades of research, the clinical positioning of these agents remains a subject of debate. While several trials suggest symptomatic improvement with ALA, long-term structural modification has not been conclusively demonstrated [9]. For benfotiamine, a strong biochemical rationale contrasts with relatively limited and short-term clinical evidence [10,11]. Given the growing emphasis on nutritional and metabolic interventions in chronic disease management, a critical reassessment of the mechanistic rationale and clinical evidence supporting ALA and benfotiamine in DPN is warranted. The present review aims to synthesize current knowledge, evaluate translational limitations, and clarify the therapeutic positioning of these agents within the framework of contemporary DPN management.

## 2. Aims, Objectives, and Rationale of the Review

Despite long-standing interest in metabolic interventions for diabetic peripheral neuropathy (DPN), the clinical positioning of nutritionally derived agents such as alpha-lipoic acid (ALA) and benfotiamine remains insufficiently clarified. While multiple reviews have addressed either oxidative stress in DPN or individual therapeutic compounds [3,12,13], few have provided an integrated, critically balanced evaluation of both agents within a unified metabolic and nutritional framework. Furthermore, prior reviews have frequently focused either on symptomatic outcomes or on mechanistic plausibility, without systematically examining the translational gap between biochemical rationale and clinically meaningful endpoints. In addition, the expanding literature on mitochondrial dysfunction, AGE–RAGE signaling, and metabolic flux modulation in diabetic complications warrants a contemporary reassessment of these interventions.

### 2.1. Aim

The primary aim of this narrative review is to critically evaluate the mechanistic basis and clinical evidence supporting alpha-lipoic acid and benfotiamine as adjunctive metabolic therapies in diabetic peripheral neuropathy.

### 2.2. Specific Objectives

The objectives of this review are:

1. To synthesize current understanding of the metabolic and oxidative pathways involved in DPN pathogenesis and to identify therapeutic targets relevant to ALA and benfotiamine.
2. To analyze preclinical evidence supporting the biological plausibility of these agents.
3. To critically appraise randomized controlled trials and long-term clinical studies evaluating symptomatic and structural outcomes.
4. To assess the strength, consistency, and limitations of the available evidence.
5. To clarify the clinical positioning of these compounds within a nutritional therapeutic context.

### 2.3. Rationale and Novelty

This review attempts to offer a perspective that differs from previous publications in the following aspects. First, it integrates oxidative stress modulation (ALA) and metabolic pathway diversion via transketolase activation (benfotiamine) into a single comparative framework, allowing for mechanistic and clinical contrast rather than isolated evaluation. Second, it emphasizes the distinction between symptomatic improvement and disease modification, a critical but often blurred concept in DPN research. Third, it evaluates these agents specifically within a nutritional and metabolic paradigm, aligning with the evolving perspective that micronutrient-derived interventions may influence the trajectory of chronic metabolic complications. Fourth, it provides a structured critical appraisal of translational limitations, including trial duration, endpoint selection, and the paucity of structural biomarkers, although without using a formal evidence-grading system (e.g., GRADE).

Given the persistent burden of DPN despite optimized glycemic control and the growing clinical use of nutraceutical and metabolic supplements, a balanced and transparent reassessment is necessary to inform evidence-based integration into clinical practice while acknowledging the limitations of the available evidence. Fifth, while alpha-lipoic acid has been extensively studied, this review provides a novel contribution by explicitly grading the evidence for symptomatic versus structural outcomes, by directly comparing ALA with a mechanistically distinct agent (benfotiamine), and by clearly stating that no trial has demonstrated nerve regeneration, a point often omitted or obscured in previous reviews.

We acknowledge that the term ‘nutritional framework’ is used here to describe the use of supplement-level metabolic modulators, not whole-diet interventions. This is a narrower interpretation than some readers might expect, but it reflects the current state of evidence: most research on ALA and benfotiamine has been conducted in the context of supplementation, not dietary patterns or essential nutrient repletion.

Positioning our work relative to existing literature, we note the following examples: previous narrative reviews have focused either on ALA alone or on benfotiamine alone, without direct comparison; others have discussed multiple nutritional interventions but without a structured grading of evidence strength.

This review does not present new experimental data, and its conclusions largely align with existing evidence syntheses. The main value added is the comparative framework, the explicit separation of outcome types, and the emphasis on the gap between mechanistic plausibility and clinical proof.

## 3. Materials and Methods

This work is a narrative review designed to provide a comprehensive, critical, and integrative synthesis of the literature on alpha-lipoic acid and benfotiamine in diabetic peripheral neuropathy. To enhance methodological transparency, a systematic approach to literature searching and data extraction was adopted, as recommended for narrative reviews aiming to minimize selection bias.

### 3.1. Literature Search Strategy

A structured literature search was performed using the PubMed/MEDLINE database covering the period from database inception up to December 2025. The search strategy was designed to identify all potentially relevant publications, employing a combination of Medical Subject Headings (MeSH) terms and free-text keywords. The following search terms were used alone and in various combinations using the Boolean operators AND and OR:

- Population/Problem: “diabetic peripheral neuropathy,” “diabetic polyneuropathy,” and “diabetic neuropathies”
- Intervention: “alpha-lipoic acid,” “thioctic acid,” “benfotiamine,” “thiamine derivatives,” and “vitamin B1”
- Mechanism/Context: “oxidative stress,” “advanced glycation end products,” “transketolase,” “metabolic therapy,” and “antioxidant therapy”

The reference lists of all included full-text articles and relevant review papers were manually screened (snowballing) to identify additional studies not captured by the initial electronic search.

The search yielded 347 records after duplicate removal. Following title/abstract screening, 89 full-text articles were assessed for eligibility, of which 47 were included in this review (clinical trials and preclinical studies). These numbers are provided for descriptive transparency only and do not imply a systematic review or meta-analysis. No formal risk-of-bias assessment (e.g., Cochrane RoB 2) or quantitative synthesis was performed.

### 3.2. Eligibility Criteria

The following criteria were used to guide study selection in a structured manner, consistent with best practices for narrative reviews, but without the formal requirements of a systematic review (e.g., dual-reviewer screening or exhaustive gray literature search). Studies were considered eligible for inclusion in this review based on the following predefined criteria, organized according to the PICO (Population, Intervention, Comparison, Outcome) framework:

*Inclusion Criteria:*

1. **Publication Type:** Peer-reviewed original research (randomized controlled trials, controlled clinical trials, long-term prospective follow-up studies, and preclinical in vivo/in vitro studies for mechanistic background). Systematic reviews and meta-analyses were not included as primary evidence sources to avoid double-counting of original studies; however, their reference lists were screened for additional primary studies (snowballing).
2. **Study Design:** For clinical evidence, randomized controlled trials (RCTs), controlled clinical trials, and long-term prospective follow-up studies were included. For mechanistic background, preclinical (in vivo and in vitro) studies and human translational research were considered.
3. **Population (P):** Studies involving human participants aged > 18 years with diagnosed Type 1 or Type 2 diabetes mellitus and confirmed diabetic peripheral neuropathy. Preclinical studies using animal models of diabetic neuropathy were also included for mechanistic rationale.
4. **Intervention (I):** Studies evaluating alpha-lipoic acid or benfotiamine as a therapeutic intervention, administered orally or intravenously, either as monotherapy or in combination, provided the effect of the specific agent could be discerned.
5. **Comparison (C):** Studies with placebo, no treatment, active comparator, or dose-comparison designs were eligible.
6. **Outcomes (O):** Studies reporting on clinical outcomes (e.g., neuropathic pain scales, Total Symptom Score [TSS]), neurophysiological outcomes (e.g., nerve conduction studies), validated symptom-based outcomes, quality of life measures, or mechanistic endpoints (e.g., markers of oxidative stress, AGEs).
7. **Language:** Publications in English.

Landmark trials of foundational importance (e.g., ALADIN, SYDNEY, NATHAN 1) were included irrespective of their publication date [4,5,6,7].

*Exclusion Criteria:*

1. Studies involving non-diabetic neuropathy populations.
2. Studies using combination therapies where the independent clinical effect of ALA or benfotiamine could not be distinguished (e.g., fixed-dose combinations with other B-vitamins without a monotherapy arm), although such studies were noted for context.
3. Studies that lacked defined neuropathy-specific endpoints.
4. Publications such as narrative opinions, commentaries, conference abstracts, or letters to the editor without primary data.
5. Articles not published in English.

### 3.3. Study Selection and Data Extraction

The study selection process was conducted in a staged manner. First, titles and abstracts of all retrieved records were screened against the eligibility criteria. Second, the full texts of potentially relevant articles were obtained and thoroughly assessed for final inclusion. Data from the included studies were extracted into a standardized electronic form. Key data points included author and year, study design, participant characteristics (type of diabetes, neuropathy severity), intervention details (agent, dose, route, duration), comparator, primary and secondary outcomes, and main findings relevant to the objectives of this review.

### 3.4. Data Synthesis and Analysis

Given the narrative design and the expected heterogeneity in study designs, populations, interventions, and outcome measures, a formal meta-analysis was not conducted. Instead, a qualitative and integrative synthesis was performed. The evidence was organized thematically, first by agent (ALA and benfotiamine) and then by the nature of the evidence (mechanistic, clinical). A comparative analysis was then undertaken, structured around key thematic axes:

- **Biological Plausibility:** The strength and consistency of the mechanistic rationale.
- **Strength and Consistency of Clinical Evidence:** The volume, quality, and reproducibility of findings from clinical trials.
- **Duration of Intervention:** The distinction between short-term (weeks) and long-term (months to years) studies.
- **Type of Outcome:** The differentiation between symptomatic improvement (patient-reported outcomes) and evidence of structural or disease-modifying effects (e.g., nerve conduction studies, morphological biomarkers).
- **Methodological Rigor:** Sample size, study design (RCT vs. open-label), and risk of bias.

To enhance the clarity and utility of the synthesis, the key findings from the comparative analysis are presented in tabular form within the Results section, summarizing the mechanistic and clinical profiles of both agents and providing a head-to-head comparison.

To avoid the risk of study repetition (double-counting of the same primary studies across different systematic reviews), this review did not extract or synthesize data from previously published systematic reviews or meta-analyses. Instead, all clinical and mechanistic conclusions were drawn directly from original research articles. Systematic reviews identified during the search were used only for hand-searching their reference lists to locate additional primary studies that might have been missed by the electronic database search. This approach ensures that each primary study contributes only once to the evidence synthesis. Consistent with the narrative review design, no formal quantitative synthesis, meta-analysis, or statistical pooling of results was performed.

### 3.5. Methodological Quality Assessment

A formal risk-of-bias assessment tool (e.g., Cochrane RoB 2) was not applied, which represents a limitation of this narrative review. However, to uphold the ‘critical’ nature of this review, we predefined a set of methodological quality indicators that were assessed for each included clinical trial. These indicators were derived from standard critical appraisal principles and included:

- Randomization method (explicitly described or not);
- Blinding (double-blind, single-blind, or open-label);
- Sample size (adequacy for primary endpoint);
- Treatment duration (short-term ≤ 12 weeks vs. long-term ≥ 1 year);
- Type of outcome measures (symptom-based scales only vs. inclusion of structural or neurophysiological endpoints);
- Use of intention-to-treat (ITT) analysis;
- Completeness of follow-up (attrition rate).

These indicators were applied qualitatively to each study during data extraction. The following limitations were explicitly noted where present: short trial duration (≤12 weeks), lack of blinding, small sample size (*n* < 100), absence of intention-to-treat analysis, reliance exclusively on symptom-based endpoints without structural measures, and high attrition rate (>20%). No summary score, quantitative meta-analytic weighting, or GRADE certainty assessment was assigned, consistent with the narrative design. Consequently, the labels used throughout this review (e.g., “well-established,” “consistent,” “limited,” “insufficient”) represent the authors’ qualitative interpretation of the volume and consistency of available studies, not statistically derived certainty levels.

## 4. Results

### 4.1. Metabolic and Molecular Basis of Diabetic Peripheral Neuropathy

**Figure 1:**
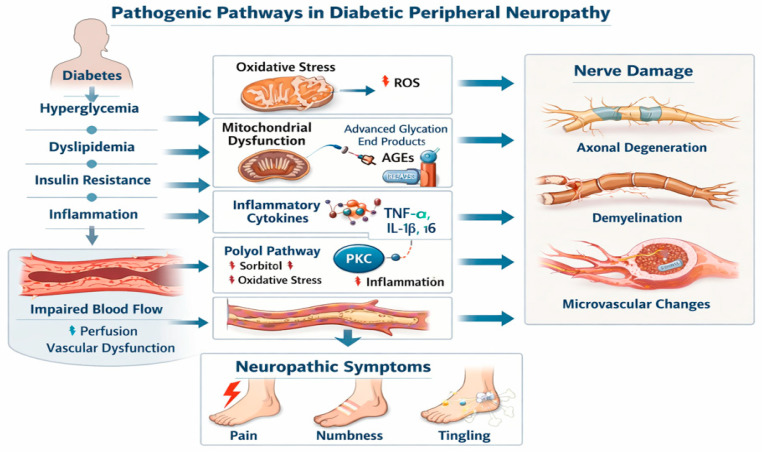
**Pathogenic pathways involved in diabetic peripheral neuropathy.** Chronic hyperglycemia induces a cascade of metabolic disturbances, including oxidative stress, mitochondrial dysfunction, activation of the polyol pathway, formation of advanced glycation end products (AGEs), and protein kinase C (PKC) signaling. These processes contribute to inflammatory activation, microvascular dysfunction, and impaired neural perfusion, ultimately leading to axonal degeneration, demyelination, and the development of neuropathic symptoms such as pain, numbness, and tingling.

DPN is the result of complex and interrelated metabolic, vascular, and inflammatory mechanisms triggered by chronic hyperglycemia. Although traditionally considered a microvascular complication, growing evidence supports the concept that DPN is primarily a metabolically driven neurodegenerative disorder with secondary vascular involvement, as shown in Figure 1 [12,14,15,16].

#### 4.1.1. Mitochondrial Dysfunction and Oxidative Stress as Central Drivers

The unifying hypothesis proposed by Brownlee identifies mitochondrial overproduction of superoxide as the initiating event in hyperglycemia-induced tissue damage [3]. Excess intracellular glucose increases flux through glycolysis and the tricarboxylic acid cycle, elevating electron donors (NADH and FADH_2_) entering the mitochondrial respiratory chain. This results in an increased mitochondrial membrane potential and partial reduction of oxygen, generating superoxide anions [3]. Reactive oxygen species (ROS) subsequently inhibit glyceraldehyde-3-phosphate dehydrogenase (GAPDH), leading to upstream accumulation of glycolytic intermediates that are redirected into four major pathogenic pathways: the polyol pathway, the advanced glycation end product (AGE) pathway, protein kinase C (PKC) activation, and the hexosamine pathway. These pathways collectively impair neuronal energy metabolism, axonal transport, and microvascular perfusion [3,17].

Oxidative stress has been consistently demonstrated in experimental and clinical DPN. Elevated markers of lipid peroxidation, reduced glutathione levels, and impaired antioxidant enzyme activity have been reported in patients with diabetic neuropathy [18,19]. Peripheral nerves are particularly vulnerable to oxidative damage due to their high metabolic demand and relatively low antioxidant capacity [18]. Mitochondrial dysfunction further contributes to impaired ATP production, axonal degeneration, and distal nerve fiber loss, with experimental studies showing altered mitochondrial morphology, decreased respiratory chain activity, and increased apoptosis in diabetic nerve tissue [18].

#### 4.1.2. Polyol Pathway Activation

Under normoglycemic conditions, only a small fraction of glucose is metabolized via aldose reductase. However, hyperglycemia increases polyol pathway flux, converting glucose to sorbitol and subsequently to fructose [17]. This process consumes NADPH, a critical cofactor required for the regeneration of reduced glutathione, thereby weakening endogenous antioxidant defenses [17]. Accumulation of sorbitol also induces osmotic stress and disrupts myoinositol metabolism, contributing to impaired nerve conduction velocity [17].

#### 4.1.3. Advanced Glycation End Products (AGEs) and RAGE Signaling

AGEs are formed through non-enzymatic glycation of proteins, lipids, and nucleic acids under chronic hyperglycemia [20]. These modifications alter protein structure and function, impair extracellular matrix integrity, and contribute to microvascular stiffening. AGEs exert pathogenic effects via two principal mechanisms: direct cross-linking of extracellular matrix proteins and binding to the receptor for advanced glycation end products (RAGE). Activation of RAGE initiates intracellular signaling cascades involving NF-κB, increased expression of pro-inflammatory cytokines, and further ROS production [20,21]. This establishes a self-amplifying inflammatory–oxidative loop that perpetuates neuronal injury. In peripheral nerves, AGE accumulation has been associated with reduced nerve conduction velocity and structural alterations of the endoneurial microvasculature [20].

#### 4.1.4. Protein Kinase C Activation and Microvascular Dysfunction

Hyperglycemia-induced diacylglycerol (DAG) accumulation activates PKC isoforms, particularly PKC-β [13]. PKC activation impairs endothelial nitric oxide synthase (eNOS) activity, reduces nitric oxide bioavailability, and increases endothelin-1 expression, leading to vasoconstriction and reduced endoneurial blood flow [13]. This microvascular dysfunction contributes to nerve hypoxia, impaired nutrient delivery, and axonal degeneration, with reduced nerve perfusion documented in both experimental models and patients with DPN [18].

#### 4.1.5. Hexosamine Pathway and Gene Expression Modulation

Excess fructose-6-phosphate is diverted into the hexosamine pathway, generating UDP-N-acetylglucosamine, which modifies transcription factors via O-linked glycosylation [17]. This alters gene expression patterns, promoting pro-inflammatory and pro-fibrotic signaling. Although less directly studied in neuropathy compared to retinopathy and nephropathy, hexosamine pathway activation contributes to the global metabolic dysregulation characteristic of diabetic complications.

#### 4.1.6. Neurotrophic Factor Deficiency and Impaired Regeneration

Chronic metabolic stress reduces levels of neurotrophic factors such as nerve growth factor (NGF) and insulin-like growth factor-1 (IGF-1), impairing neuronal survival and regenerative capacity [19,21,22,23,24]. This reduced neurotrophic support exacerbates distal axonal degeneration and delays repair mechanisms, contributing to the progressive and largely irreversible nature of established DPN.

Collectively, these mechanisms demonstrate that DPN is driven by intertwined oxidative, metabolic, inflammatory, and microvascular disturbances, with oxidative stress and metabolic flux dysregulation occupying central positions within this network. This provides the mechanistic rationale for metabolic-targeted interventions such as alpha-lipoic acid and benfotiamine.

**Conceptual Framework: Distinguishing Symptomatic, Structural, and Metabolic Effects in DPN Trials**

To interpret the clinical evidence for ALA and benfotiamine correctly, it is essential to distinguish three types of therapeutic effects:

- Metabolic effects: Changes in biochemical pathways (e.g., reduced oxidative stress, increased transketolase activity, decreased AGE formation). These are measured in preclinical or translational studies and may not directly correlate with clinical improvement.
- Symptomatic effects: Patient-reported outcomes such as pain, paresthesia, numbness, and Total Symptom Score (TSS). These are the primary endpoints in most DPN trials.
- Structural effects: Objective measures of nerve integrity, including nerve conduction velocity, intraepidermal nerve fiber density (IENFD), and corneal confocal microscopy (CCM). Improvement in these measures would indicate nerve regeneration or repair, which has not been demonstrated for either agent.

The available evidence for ALA supports consistent symptomatic improvement and possible functional stabilization (as measured by the Neuropathy Impairment Score—Lower Limbs in the NATHAN 1 trial), but not structural regeneration. For benfotiamine, only short-term symptomatic data exist, with no evidence of structural or durable metabolic effects in humans. This distinction is maintained throughout the following sections.

### 4.2. Alpha-Lipoic Acid: Biochemical Rationale and Clinical Evidence

#### 4.2.1. Biochemical and Pharmacological Properties

Alpha-lipoic acid, also known as thioctic acid, is a naturally occurring disulfide compound synthesized in mitochondria, where it functions as an essential cofactor for pyruvate dehydrogenase and α-ketoglutarate dehydrogenase complexes [25]. In its reduced form, dihydrolipoic acid (DHLA), it exerts potent antioxidant activity. ALA possesses several biochemical properties relevant to diabetic peripheral neuropathy: direct scavenging of reactive oxygen species (ROS), including hydroxyl radicals and singlet oxygen [26]; regeneration of endogenous antioxidants such as glutathione, vitamin C, and vitamin E [27]; chelation of transition metals, limiting Fenton chemistry-mediated oxidative damage [28]; modulation of redox-sensitive transcription factors, including NF-κB [29]; and improvement of endothelial function and microvascular blood flow [30].

Unlike many antioxidants, ALA is both water- and lipid-soluble, allowing broad intracellular distribution, including within mitochondrial compartments. Given the central role of mitochondrial oxidative stress in DPN pathogenesis, this pharmacokinetic characteristic is mechanistically relevant [3,18]. In addition to its antioxidant effects, ALA may influence glucose metabolism. Experimental and clinical data suggest improved insulin sensitivity and enhanced glucose uptake, potentially via activation of AMP-activated protein kinase (AMPK) pathways [30,31]. These metabolic effects may contribute indirectly to neuropathic improvement.

**Figure 2:**
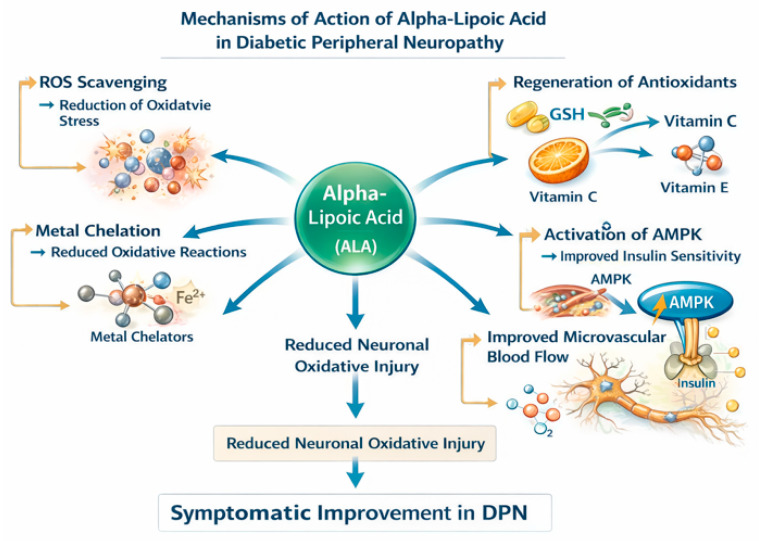
Mechanisms of action of alpha-lipoic acid in diabetic peripheral neuropathy. Alpha-lipoic acid exerts multiple biochemical and metabolic effects relevant to the pathogenesis of diabetic peripheral neuropathy. These include direct scavenging of reactive oxygen species, regeneration of endogenous antioxidants such as glutathione and vitamins C and E, chelation of transition metals, activation of AMP-activated protein kinase (AMPK) with improved insulin sensitivity, and enhancement of microvascular blood flow. Together, these mechanisms contribute to reduced oxidative neuronal injury and symptomatic improvement in diabetic peripheral neuropathy.

The principal antioxidant and metabolic mechanisms through which alpha-lipoic acid may exert therapeutic effects in diabetic peripheral neuropathy are illustrated in Figure 2.

#### 4.2.2. Evidence from Randomized Controlled Trials

ALA has been evaluated in several landmark randomized controlled trials (RCTs) in patients with symptomatic diabetic polyneuropathy.

The **ALADIN I** study was a multicenter, randomized, placebo-controlled trial evaluating intravenous ALA (100–600 mg/day) for three weeks in 328 patients with symptomatic DPN [4]. The 600 mg dose demonstrated significant improvement in the Total Symptom Score (TSS) compared with placebo. **ALADIN III** extended the investigation to oral administration over seven months and confirmed symptomatic improvement, though gastrointestinal adverse events were more frequent at higher doses [5].

The **SYDNEY** trial evaluated intravenous ALA 600 mg/day for three weeks in 120 patients and demonstrated significant reductions in neuropathic symptoms and the Neuropathy Impairment Score (NIS) compared with placebo [6]. The **SYDNEY 2** trial assessed oral ALA (600–1800 mg/day) for five weeks in 181 patients [7]. The 600 mg/day dose achieved the best efficacy–tolerability balance, with significant improvement in TSS and acceptable adverse event rates.

The NATHAN 1 study represents the most important long-term investigation of oral ALA [32]. In this four-year randomized, double-blind, placebo-controlled trial involving 460 patients with mild-to-moderate DPN, ALA 600 mg/day showed a marginal improvement in the primary composite endpoint (NIS-LL) compared with placebo, with a *p*-value of 0.05 that reached conventional statistical significance only in certain sensitivity analyses. No significant differences were observed in nerve conduction studies or in most secondary neurophysiological endpoints. Consequently, the trial provides limited evidence for possible stabilization of neuropathic deficits but does not demonstrate structural regeneration or a definitive disease-modifying effect. The clinical meaningfulness of the observed changes remains debated.

#### 4.2.3. Meta-Analytical Evidence

A meta-analysis by Mijnhout et al. evaluated intravenous ALA and reported significant short-term improvement in neuropathic symptoms [33]. However, heterogeneity across trials was noted. More recently, updated systematic reviews indicate that oral ALA at doses of 600–1800 mg/day may provide modest symptomatic benefit, particularly in early-stage neuropathy, but evidence for long-term disease modification remains limited [34]. Importantly, improvements are most consistently observed in symptom-based scales (TSS, NIS), whereas objective neurophysiological measures show smaller or inconsistent effects.

#### 4.2.4. Safety and Tolerability

ALA is generally well tolerated. The most common adverse effects include nausea, vomiting, and dyspepsia, particularly at doses exceeding 600 mg/day [7]. Severe adverse events are rare. Because ALA may modestly improve insulin sensitivity, caution is advised in patients receiving intensive glucose-lowering therapy due to a potential, though uncommon, risk of hypoglycemia.

#### 4.2.5. Critical Appraisal

While ALA demonstrates consistent short-term symptomatic benefit across multiple RCTs, several limitations must be acknowledged. Many trials are of short duration (3–5 weeks). Structural endpoints such as intraepidermal nerve fiber density or corneal confocal microscopy were not routinely assessed. The long-term NATHAN 1 trial reported a marginal effect on the primary composite endpoint (NIS-LL, *p* = 0.05) without significant improvements in nerve conduction studies; therefore, it provides only weak evidence for possible stabilization and no evidence for reversal of neuropathy [32]. Furthermore, most studies included patients with mild-to-moderate neuropathy, limiting generalizability to advanced disease.

Therefore, the current evidence supports ALA as an adjunctive symptomatic therapy for short-term relief (weeks to months). Any disease-modifying or neuroregenerative effect remains unproven. The NATHAN 1 trial [32] reported a marginal effect on the primary composite endpoint (NIS-LL, *p* = 0.05) that reached conventional statistical significance only in certain sensitivity analyses; this finding should be interpreted cautiously and does not constitute robust evidence for disease modification.

Important note on outcome measures: Across all included clinical trials for both ALA and benfotiamine, the primary evidence for efficacy is based on symptom-based scales (e.g., Total Symptom Score, Neuropathy Impairment Score, and visual analog scales for pain). No study has demonstrated unequivocal nerve regeneration using validated structural biomarkers such as intraepidermal nerve fiber density (IENFD) or corneal confocal microscopy (CCM). Nerve conduction studies, when reported, showed inconsistent or no significant improvement. Therefore, the current evidence supports symptomatic relief and possible functional stabilization, but not structural repair or regeneration of damaged nerve fibers.

### 4.3. Benfotiamine: Metabolic Modulation and Clinical Evidence

#### 4.3.1. Thiamine Metabolism and Mechanistic Rationale

Benfotiamine is a lipid-soluble S-acyl derivative of thiamine (vitamin B1) developed to overcome the limited bioavailability of water-soluble thiamine salts [35]. After intestinal absorption, benfotiamine is dephosphorylated and converted into biologically active thiamine, resulting in higher intracellular thiamine levels compared to conventional supplementation [36]. Thiamine functions as a cofactor for several key enzymes in glucose metabolism, including transketolase (pentose phosphate pathway), pyruvate dehydrogenase, and α-ketoglutarate dehydrogenase.

In the context of chronic hyperglycemia, insufficient transketolase activity contributes to the accumulation of glycolytic intermediates upstream of glyceraldehyde-3-phosphate dehydrogenase inhibition. These intermediates are diverted into the polyol pathway, AGE formation, PKC activation, and hexosamine flux [3,17]. Experimental studies have demonstrated that benfotiamine increases transketolase activity and reduces flux through these three major hyperglycemia-induced damage pathways [8]. In diabetic animal models, benfotiamine prevented AGE accumulation, reduced oxidative stress markers, and improved nerve conduction parameters [37,38]. Hammes et al. showed that benfotiamine blocked hyperglycemia-induced damage pathways in experimental diabetic retinopathy, supporting the concept of metabolic flux redirection as a therapeutic strategy [8]. Although retinopathy and neuropathy are distinct complications, they share common upstream metabolic disturbances. Thus, benfotiamine offers strong biochemical plausibility by targeting hyperglycemia-induced metabolic dysregulation at an upstream level.

#### 4.3.2. Preclinical Evidence in Neuropathy Models

In diabetic rodent models, benfotiamine supplementation improved nerve conduction velocity and reduced markers of glycoxidative stress [37]. Reduction in neural imidazole-type AGE formation and normalization of oxidative stress markers were reported in experimental settings [38]. However, while preclinical data are mechanistically consistent, translation to human neuropathy outcomes remains complex, as animal models often represent early or induced neuropathy states and may not capture the chronic, multifactorial nature of human DPN.

#### 4.3.3. Clinical Trials in Diabetic Peripheral Neuropathy

Clinical evidence for benfotiamine in DPN is more limited and generally of shorter duration compared to ALA. Early combination studies, such as that by Stracke et al., evaluated a benfotiamine–vitamin B combination in patients with diabetic polyneuropathy and reported improvement in neuropathic symptoms over a short treatment period [10]. However, such combination therapy limits attribution of effects specifically to benfotiamine. Winkler et al. investigated different benfotiamine dosage regimens in painful diabetic neuropathy and observed dose-dependent symptomatic improvement over three weeks [39]. These trials were small and short-term.

The **BENDIP** study (Benfotiamine in Diabetic Polyneuropathy) was a randomized, double-blind, placebo-controlled trial evaluating benfotiamine 300–600 mg/day over six weeks [11]. The study reported improvement in neuropathic symptom scores, particularly at higher doses. However, several limitations must be noted, including its short duration (six weeks), reliance on symptom-based endpoints, absence of structural or long-term functional measures, and limited sample size. Importantly, no large-scale, long-duration trial comparable to NATHAN 1 exists for benfotiamine in DPN.

#### 4.3.4. Clinical Interpretation and Limitations

Despite a compelling biochemical rationale, human clinical evidence for benfotiamine remains less robust than that for ALA. Key limitations include predominantly short-duration studies (≤6–12 weeks), reliance on symptom-based scales without structural biomarkers, frequent use of combination therapy with other B vitamins, and limited long-term neurophysiological data. Moreover, most trials enrolled patients without documented thiamine deficiency. Whether benfotiamine provides greater benefit in subgroups with relative intracellular thiamine insufficiency remains unclear. While vitamin B supplementation is clearly indicated in documented deficiency states, extrapolation to routine use in unselected DPN populations lacks strong long-term validation [40].

Benfotiamine represents a metabolically rational intervention targeting upstream glucose toxicity mechanisms. However, current clinical evidence supports, at most, modest short-term symptomatic benefit. In contrast to ALA, evidence for long-term stabilization or disease modification is insufficient. The gap between strong biochemical plausibility and limited clinical validation highlights the translational challenges inherent in metabolic therapy for chronic diabetic complications.

Thus, based on current data (which are limited to short-duration, symptom-only trials), benfotiamine cannot be recommended as a disease-modifying therapy for DPN, nor can it be recommended for routine clinical use outside of documented thiamine deficiency. The strong biochemical rationale does not compensate for the absence of robust clinical validation. Any consideration of benfotiamine should be accompanied by clear patient counseling that no large, long-term trial has demonstrated structural benefit or disease modification.

### 4.4. Comparative Mechanistic and Clinical Analysis

To facilitate a direct and structured comparison of the two agents, the key characteristics of alpha-lipoic acid and benfotiamine are summarized in the following tables. Table 1 provides a comparative overview of their mechanistic profiles, highlighting their distinct but complementary targets within the DPN pathogenic cascade.

The following tables summarize the authors’ qualitative interpretation of the evidence. Because no formal risk-of-bias assessment or evidence-grading framework (e.g., GRADE) was applied, all descriptive labels (including but not limited to ‘well-established’, ‘consistent’, ‘limited’, ‘insufficient’, ‘absent’, ‘more mature’, etc.) represent the authors’ subjective judgment based on the volume, consistency, and duration of available studies. These labels are not statistically derived certainty levels and should be interpreted as heuristic summaries only.

As Table 1 illustrates, ALA acts primarily as a broad-spectrum antioxidant and redox modulator, intervening downstream of initial metabolic stress. In contrast, benfotiamine targets the upstream metabolic flux, aiming to prevent the formation of damaging intermediates. This conceptual distinction is important for understanding their potential roles in therapy.

Table 2 summarizes the clinical evidence base for each agent, highlighting the volume, duration, and key outcomes of the major trials.

The data synthesized in Table 2 clearly demonstrate the disparity in the evidence bases. ALA is supported by a larger number of robust, long-term trials, while the evidence for benfotiamine, although suggestive, is considerably less mature.

Finally, Table 3 provides a synthesized overview of the comparative evidence and proposes a current therapeutic positioning for each agent based on the available data.

As Table 3 consolidates, while both agents are mechanistically plausible, ALA has a more established clinical profile. Benfotiamine remains a promising intervention for disease modification in DPN, highlighting a critical gap between its strong biochemical rationale and its clinical validation.

To enhance scholarly transparency and allow readers to independently evaluate the evidence base, Table 1 and Table 2 summarize the design, key findings, adverse events, and methodological limitations of the major clinical trials of alpha-lipoic acid and benfotiamine, respectively (Table 4 and Table 5).

## 5. Discussion

Diabetic peripheral neuropathy remains a major clinical challenge despite advances in glycemic management and cardiovascular risk control. Before interpreting the following discussion, readers should be aware that this is a narrative review, not a systematic review or meta-analysis. The evidence synthesis is qualitative, and the conclusions reflect the authors’ interpretation of the available literature, which is limited to PubMed and English-language publications.

The persistent progression of neuropathy in many patients underscores the multifactorial and metabolically complex nature of this complication [1,2]. The present review highlights that both alpha-lipoic acid (ALA) and benfotiamine are grounded in biologically plausible mechanisms targeting central components of hyperglycemia-induced injury. However, as systematically outlined in the comparative tables (Table 1, Table 2 and Table 3), the strength of translational evidence differs substantially between the two agents.

It is important to note that no direct head-to-head randomized controlled trials comparing alpha-lipoic acid and benfotiamine exist, and no statistical tests can be applied to compare their efficacy indirectly due to heterogeneity in study designs, populations, outcomes, and durations. Therefore, any comparison in this discussion is qualitative and based on the volume, consistency, and methodological quality of available evidence, not on statistical superiority.

### 5.1. Clinical Applications and Practical Considerations

The following subsections describe how alpha-lipoic acid and benfotiamine have been used in published studies. These descriptions are based exclusively on the available literature and should not be interpreted as formal clinical recommendations or practice guidelines. Given the narrative nature of this review and the absence of systematic evidence grading or guideline-level sources, no definitive clinical guidance can be provided.

#### 5.1.1. Alpha-Lipoic Acid in Clinical Practice

Based on the available evidence (which is limited to a narrative review without formal bias assessment), some clinicians have used ALA as an adjunctive therapy for patients with symptomatic DPN, particularly those with early-to-moderate disease. The most frequently studied regimen is oral ALA at 600 mg once daily, which showed the best efficacy-tolerability balance in the SYDNEY 2 trial [7] and was also used in the long-term NATHAN 1 trial [32].

Potential contexts in which ALA has been considered in clinical practice include patients with mild-to-moderate neuropathic symptoms who have inadequate response or intolerance to first-line pharmacological agents [2,20], individuals seeking additional symptom control, and patients in early stages of DPN, where metabolic interventions might theoretically slow progression. However, it is important to note that these are not formal recommendations, as the evidence base does not support definitive guidance. However, it must be emphasized that no clinical trial has demonstrated that ALA slows disease progression or prevents structural nerve damage. The theoretical rationale for slowing progression remains unvalidated.

In the published trials, the onset of symptomatic benefit was typically observed within 3–5 weeks [4,6,7]. Some investigators have continued treatment for 3–6 months to assess response, but no standardized protocol has been validated. Patients should be informed that the primary evidence supports symptomatic improvement and possible stabilization, not reversal of established nerve damage, and that these conclusions are based on a narrative review with methodological limitations.

#### 5.1.2. Benfotiamine in Clinical Practice

Despite a strong biochemical rationale [8,35,36,37,38], the clinical evidence base for benfotiamine in DPN is very limited. No large-scale, long-term (≥1 year) randomized controlled trial has been published. The available studies are of short duration (≤6–12 weeks), rely exclusively on symptom-based endpoints, and lack structural or neurophysiological outcome measures [10,11,39]. Consequently, benfotiamine cannot be recommended as a disease-modifying therapy for DPN. Its use, if any, should be restricted to patients with documented or suspected thiamine deficiency or as part of an experimental nutritional strategy with appropriate patient counseling about the absence of robust evidence. However, benfotiamine may have practical utility in specific clinical contexts:

It is important to reiterate that these potential contexts are based on mechanistic reasoning and extrapolation from preclinical data, not on clinical trial evidence demonstrating benefit for neuropathic outcomes. In all cases, patients should be clearly informed that benfotiamine has not been shown to improve structural nerve measures or to modify disease progression. The decision to use benfotiamine should be made with full transparency about the lack of robust evidence.

The clinical application of benfotiamine requires considerable caution. Despite a strong biochemical rationale [8,35,36,37,38], the clinical evidence base for benfotiamine in DPN is very limited. No large-scale, long-term (≥1 year) randomized controlled trial has been published. The available studies are of short duration (≤6–12 weeks), rely exclusively on symptom-based endpoints, and lack structural or neurophysiological outcome measures [10,11,39]. Consequently, based on this narrative review, there is insufficient evidence to recommend benfotiamine as a disease-modifying therapy for DPN.

Some authors have suggested potential utility in specific contexts based on mechanistic reasoning rather than clinical trial evidence. These include patients with documented or suspected thiamine deficiency (given the high prevalence of low thiamine levels in diabetes [41] and the superior bioavailability of benfotiamine over conventional thiamine [35,36]), as part of a broader nutritional strategy or in early metabolic dysregulation before irreversible structural damage has occurred. However, it must be emphasized that these suggestions are hypothetical and not supported by clinical outcome data.

The dosage used in published trials was typically 300–600 mg daily [11]. Patients considering benfotiamine should be clearly informed that the evidence supports, at most, modest short-term symptomatic benefit and that no trial has demonstrated structural improvement or disease modification. Given the absence of robust evidence, no formal recommendation for or against routine use can be made.

### 5.2. Comparison with Other Nutritional Interventions

Other nutritional interventions have been investigated in DPN, including acetyl-L-carnitine, vitamin D, vitamin E, and omega-3 fatty acids. Within the limits of this narrative review, ALA appears to have a relatively larger and more consistent evidence base for symptomatic improvement compared to these other agents, although direct comparisons are lacking [42]. Vitamin D deficiency is prevalent in diabetic populations and has been associated with neuropathic pain, but interventional trials have yielded mixed results [43]. Compared to these agents, ALA has the most robust evidence base for symptomatic improvement in DPN, while benfotiamine is distinguished by its specific mechanism targeting transketolase activation and metabolic flux diversion. Head-to-head comparative trials between these nutritional interventions are lacking and represent an important area for future research.

It should be noted that all these agents are used as supplements, not as part of dietary patterns. A truly nutritional framework would also consider whole-diet approaches (e.g., the Mediterranean diet), which are beyond the scope of this review.

### 5.3. Translational Gap: Why Mechanistic Plausibility Does Not Guarantee Clinical Efficacy

The discrepancy between strong metabolic rationale and modest clinical outcomes raises important translational considerations. Human DPN develops over years or decades, and short-term metabolic correction may be insufficient. Advanced axonal loss may not be recoverable through metabolic modulation alone. DPN is influenced by glycemic variability, dyslipidemia, obesity, inflammation, and genetic susceptibility, contributing to its heterogeneity [1]. Furthermore, interventions may be more effective in early or preclinical neuropathy stages, a population underrepresented in trials. These factors may partially explain why upstream metabolic interventions such as benfotiamine demonstrate stronger effects in animal models than in chronic human neuropathy.

**Current Evidence on Benfotiamine in Diabetic Peripheral Neuropathy: Mechanistic Rationale vs. Clinical Outcomes *“The following mechanistic and pharmacokinetic observations, while scientifically valid, do not compensate for the absence of robust clinical trial data. They should not be interpreted as evidence of clinical efficacy.”***

Although the current clinical evidence for benfotiamine in DPN is limited compared with that of alpha-lipoic acid, the mechanistic rationale based on thiamine deficiency in diabetes, enhanced bioavailability, and transketolase activation is biologically plausible. However, this plausibility does not translate into reproducible clinical benefit, as discussed below.

Epidemiological studies have documented that plasma thiamine concentrations in individuals with type 1 and type 2 diabetes are approximately 24–25% of those observed in healthy individuals, attributed to increased renal clearance and increased metabolic utilization. This observation indicates a relative thiamine deficiency in diabetes, which provides a biochemical rationale for thiamine supplementation. However, it does not constitute evidence that benfotiamine improves neuropathic outcomes. Benfotiamine, a lipid-soluble thiamine derivative, was developed specifically to overcome the pharmacokinetic limitations of conventional thiamine supplementation. Compared with thiamine salts, benfotiamine demonstrates substantially improved bioavailability, with significantly higher intracellular concentrations of thiamine metabolites after oral administration. Pharmacokinetic studies have shown that plasma levels of thiamine monophosphate and blood concentrations of thiamine diphosphate are markedly higher following benfotiamine administration compared with equivalent doses of thiamine hydrochloride, indicating enhanced cellular availability of the active cofactor.

Preclinical studies have shown that benfotiamine increases transketolase activity, which may divert glycolytic intermediates away from the polyol pathway, AGE formation, and PKC activation. While these mechanisms are relevant to DPN pathogenesis, their translation to clinical benefit in humans has not been robustly demonstrated. Beyond its metabolic effects, benfotiamine may also contribute indirectly to reducing oxidative stress, a central driver of diabetic microvascular complications. Elevated oxidative stress markers have been consistently reported in patients with diabetic neuropathy, and the capacity of benfotiamine to reduce upstream metabolic substrates involved in ROS generation provides a plausible neuroprotective mechanism.

**Figure 3:**
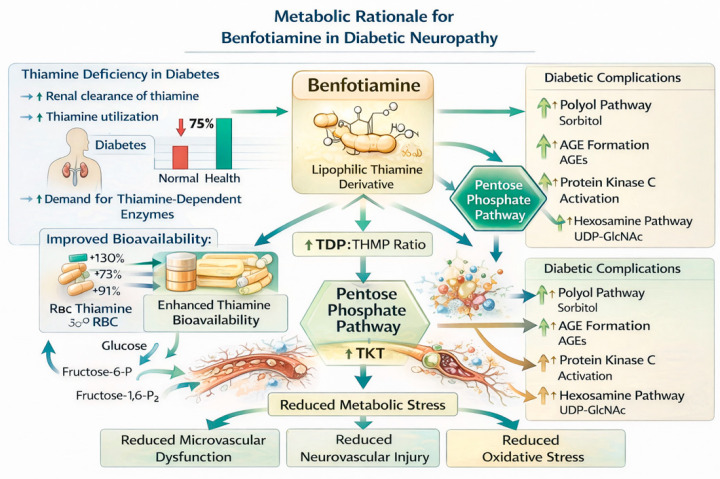
Metabolic rationale for benfotiamine in diabetic neuropathy.

Short-term clinical studies have reported symptomatic improvement with benfotiamine, often in combination with other B vitamins. However, due to the short duration, lack of structural endpoints, and combination design, these studies cannot establish a causal effect specific to benfotiamine, nor do they provide evidence for disease modification. In summary, the mechanistic rationale for benfotiamine is biologically plausible, but the clinical evidence remains insufficient to support any recommendation for routine use in DPN. No large, long-term randomized controlled trial with structural or neurophysiological endpoints has been published. The available short-term studies do not demonstrate reproducible, clinically meaningful benefit. Consequently, benfotiamine should be considered an investigational agent only, and its use outside of documented thiamine deficiency is not supported by current evidence. This positions benfotiamine as a biologically plausible adjunctive intervention that warrants further investigation in large, long-term clinical trials incorporating structural and biomarker-based endpoints, Figure 3.

Benfotiamine enhances intracellular thiamine availability and increases transketolase activity within the pentose phosphate pathway. This metabolic shift diverts excess glycolytic intermediates away from pathogenic pathways involved in diabetic complications, including the polyol pathway, advanced glycation end product formation, protein kinase C activation, and the hexosamine pathway. By reducing metabolic stress, oxidative injury, and microvascular dysfunction, benfotiamine may contribute to neurovascular protection in diabetic peripheral neuropathy.

#### Comparison with Previously Published Reviews

Several narrative reviews have previously addressed ALA or benfotiamine in DPN, e.g., [12,34,40]. However, the present review differs in four key respects: (i) it provides a direct head-to-head comparison of two mechanistically distinct agents within a single framework; (ii) it explicitly distinguishes symptomatic, structural, and metabolic effects; (iii) it systematically grades the strength of clinical evidence using predefined criteria (Table 2 and Table 3); and (iv) it clearly states the absence of nerve regeneration for both agents, which is often not emphasized elsewhere. To our knowledge, no previous review has integrated these elements.

### 5.4. Limitations of the Available Evidence

#### Limitations of This Review

This review has several limitations inherent to its narrative design. First, because this narrative review included multiple study designs (randomized trials, controlled clinical trials, preclinical experiments, and human translational studies) with different inherent biases and quality criteria, it was not possible to apply a single formal risk-of-bias tool (e.g., Cochrane RoB 2 for RCTs) uniformly across all evidence. Consequently, no quantitative or standardized bias assessment was performed. Methodological limitations of individual studies (e.g., short duration, lack of blinding, small sample size) were described qualitatively during synthesis, but the mixing of study designs precludes any overall measure of bias.

Second, only the PubMed/MEDLINE database was searched. No other databases (e.g., EMBASE, Cochrane Library, Web of Science) were consulted. Additionally, only English-language publications were included. These restrictions may have introduced database and language bias, and relevant studies published in other databases or languages may have been missed. Therefore, this review may not be fully comprehensive. Third, publication bias cannot be excluded, as studies with negative or null findings are less likely to be published and may be underrepresented in the literature. Fourth, the narrative synthesis approach, while allowing for integrative interpretation, does not provide quantitative effect estimates.

Given these limitations, the conclusions of this review should be interpreted as qualitative and hypothesis-generating rather than definitive. The structured search strategy and transparent reporting are intended to increase transparency, but they cannot fully compensate for the absence of a comprehensive database search or formal bias assessment.

All included trials relied predominantly on symptom-based outcome measures. None provided direct evidence of nerve regeneration (e.g., increased intraepidermal nerve fiber density or improved corneal nerve fiber morphology). Consequently, this review cannot draw conclusions about structural repair; it only summarizes effects on symptoms and, for ALA, possible functional stabilization.

Additionally, the comparative judgments presented in Table 1, Table 2 and Table 3 (e.g., ‘strong’, ‘moderate’, ‘weak’) are qualitative interpretations by the authors, not derived from a formal evidence-grading framework such as GRADE. Readers should interpret these labels as descriptive summaries of the volume and consistency of available studies, not as statistically validated certainty levels.

The ‘nutritional therapeutic framework’ mentioned in the title and throughout the manuscript refers specifically to the use of isolated supplement compounds, not to whole-diet interventions, dietary patterns, or essential nutrient deficiency correction. Readers should not interpret this review as a comprehensive guide to diabetes nutrition therapy, which remains centered on glycemic control through diet and lifestyle. The term is used heuristically to distinguish these metabolic modulators from conventional pharmaceuticals.

A major limitation is the exclusive reliance on the PubMed/MEDLINE database. No other databases (EMBASE, Cochrane Central, Web of Science, Scopus) were searched. Additionally, only English-language publications were included. These restrictions may have introduced database and language bias; relevant studies published in other databases (e.g., CENTRAL) or languages (e.g., German, Chinese, Russian) may have been missed. Therefore, this review is not fully comprehensive and may not capture all available evidence.

Publication bias cannot be excluded. Studies with negative or null findings are less likely to be published and may be underrepresented. The preponderance of positive short-term trials for both agents may reflect this bias.

The narrative synthesis approach, while allowing for integrative interpretation, does not provide quantitative effect estimates (e.g., mean differences, standardized effect sizes, numbers needed to treat).

Several limitations of the current evidence base must be acknowledged. Trial Duration: Many ALA and benfotiamine studies are short-term (3–12 weeks), limiting conclusions regarding long-term disease modification. Endpoint Selection: Most trials rely on symptom-based scales, while objective structural endpoints are rarely used. Heterogeneity: Differences in neuropathy severity, diabetes duration, and metabolic control complicate comparison across studies. Combination Therapies: Several benfotiamine studies involve concomitant B-vitamin supplementation, obscuring independent effects [10,39]. Publication Bias: Positive symptomatic outcomes may be preferentially published. Lack of Biomarker-Guided Stratification: Few studies assess oxidative stress markers, AGE burden, or thiamine status to identify potential responders. These limitations emphasize that current evidence supports adjunctive use but does not establish definitive disease-modifying capacity.

### 5.5. Future Directions

Future investigations should prioritize several key areas:

1. **Long-Term Randomized Controlled Trials:** Studies of ≥2–3 years’ duration evaluating structural endpoints (IENFD, CCM) are urgently needed, particularly for benfotiamine.
2. **Standardized Outcome Measures:** The adoption of core outcome sets for DPN trials would facilitate meta-analysis and cross-study comparisons. Regulatory agencies and academic consortia should work toward harmonizing endpoint selection.
3. **Biomarker-Guided Stratification:** Assessment of oxidative stress indices, AGE levels, mitochondrial function, and thiamine status [41] may identify subgroups most likely to benefit. Pharmacogenomic approaches could also identify genetic determinants of treatment response.
4. **Early Intervention Models:** Investigating metabolic therapy in early or preclinical neuropathy (e.g., patients with impaired glucose tolerance or recently diagnosed diabetes) may clarify preventive potential before irreversible structural damage occurs.
5. **Combination Metabolic Approaches:** Given the multifactorial pathogenesis of DPN, multi-target strategies combining redox modulation (ALA), metabolic correction (benfotiamine), and anti-inflammatory mechanisms warrant exploration in factorial design trials.
6. **Integration with Nutritional Optimization:** Broader dietary and micronutrient interventions should be studied in conjunction with targeted supplementation to assess synergistic effects. Mediterranean diet patterns, for example, may enhance the effects of metabolic modulators.
7. **Wearable Technology Integration:** Continuous glucose monitoring and actigraphy could provide objective measures of glycemic variability and physical activity as covariates or surrogate endpoints in clinical trials.
8. **Health Economics Evaluation:** Healthcare utilization, quality-adjusted life years, and cost-effectiveness should be assessed to inform reimbursement decisions and guideline recommendations.
9. **Epigenetic and Transcriptomic Studies:** Investigate whether metabolic interventions influence DNA methylation patterns, microRNA expression, or gene expression profiles in accessible tissues (e.g., peripheral blood mononuclear cells) as potential biomarkers of response.

Addressing these areas will be essential to determine whether metabolic interventions can meaningfully alter the trajectory of diabetic neuropathy rather than merely alleviate symptoms.

#### 5.5.1. Chemical Structure, Metabolites, and Natural Sources of Alpha-Lipoic Acid

Alpha-lipoic acid (ALA, also known as thioctic acid) is an eight-carbon fatty acid containing a terminal dithiolane ring with a disulfide bridge between C6 and C8. Its IUPAC name is 5-(1,2-dithiolan-3-yl)pentanoic acid. ALA exists as two enantiomers: the naturally occurring R-(+)-lipoic acid (R-ALA) and the synthetic S-(−)-lipoic acid (S-ALA). Only R-ALA is endogenously synthesized in mammalian mitochondria and serves as a covalently bound cofactor for multienzyme complexes, including pyruvate dehydrogenase, α-ketoglutarate dehydrogenase, and the glycine cleavage system [43].

Upon cellular uptake, ALA is enzymatically reduced to dihydrolipoic acid (DHLA) by mitochondrial and cytosolic reductases, primarily lipoamide dehydrogenase and thioredoxin reductase. DHLA contains two free thiol groups and exhibits greater antioxidant capacity than the parent compound, including direct scavenging of hydroxyl radicals, peroxyl radicals, hypochlorous acid, and singlet oxygen, as well as regeneration of glutathione, vitamin C, and vitamin E from their oxidized forms. Both ALA and DHLA can chelate transition metals such as iron, copper, and mercury, thereby inhibiting Fenton chemistry. DHLA is further metabolized via β-oxidation to bis-nor-dihydrolipoic acid and tetranor-dihydrolipoic acid, which are excreted in urine [44].

For medicinal chemistry and structure-activity relationship (SAR) studies, the dithiolane ring is essential for antioxidant activity, while the carboxylic acid side chain influences mitochondrial targeting and pharmacokinetics. The disulfide bridge in the dithiolane ring undergoes reversible reduction to dithiol, enabling redox cycling. Substitutions on the dithiolane ring or side chain generally reduce activity, whereas esterification of the carboxyl group (e.g., lipoic acid esters) can enhance bioavailability but may alter tissue distribution. R-ALA is more biologically active than S-ALA, as the S-enantiomer is less efficiently reduced to DHLA by cellular reductases. These structural features provide a basis for designing novel synthetic analogs with improved metabolic stability, tissue selectivity, or reduced gastrointestinal adverse effects [45].

#### 5.5.2. Recent Evidence

Furthermore, although the present review aimed to include recent evidence, two key synthesized sources published in 2023 and 2024, a meta-analysis by Hsieh et al. (2023) [46] and a Cochrane review by Baicus et al. (2024) [47], were not incorporated into the primary evidence synthesis due to the predefined cut-off date of the literature search. The meta-analysis by Hsieh et al. concluded that oral ALA improves sensory symptoms (Total Symptom Score) but does not produce favorable results for nerve conduction studies or vibration perception thresholds. The Cochrane review by Baicus et al. (2024) [47] found that ALA probably has little or no effect on neuropathic symptoms at six months.

## 6. Conclusions

Diabetic peripheral neuropathy is driven by complex metabolic and oxidative mechanisms that extend beyond glycemic control alone. Nutritionally derived metabolic modulators such as alpha-lipoic acid and benfotiamine target key components of this pathogenic cascade.

Based on the available literature (which has significant limitations: single database, English-only, no formal bias assessment, and potential publication bias), the following conclusions are offered as hypothesis-generating, not as definitive clinical guidance.

For alpha-lipoic acid: Short-term (3–5 weeks) symptomatic improvement is supported by multiple randomized trials [4,5,6,7]. However, the long-term NATHAN 1 trial [32] reported only a marginal, borderline significant effect on the composite NIS-LL score (*p* = 0.05) with no improvement in nerve conduction studies. A recent Cochrane review [47] found that ALA probably has little or no effect on neuropathic symptoms at six months. No study has demonstrated structural nerve regeneration or a definitive disease-modifying effect. Therefore, the evidence for any long-term benefit (symptomatic or structural) is weak.

Benfotiamine has a strong biochemical rationale, but clinical evidence remains limited to short-term, symptom-based studies without structural validation [8,10,11,39]. Therefore, its clinical utility cannot be recommended outside of specific contexts such as documented thiamine deficiency.

Given the methodological limitations of this review (single database, English-only, no formal bias assessment), these conclusions should be interpreted as hypothesis-generating and supportive of adjunctive use, not as definitive proof of disease modification. Future research should prioritize long-term randomized trials with structural endpoints and biomarker-based stratification.

## Figures and Tables

**Table 1:** Comparative mechanistic profile of ALA and benfotiamine.

Feature	Alpha-Lipoic Acid (ALA)	Benfotiamine
**Primary Mechanism of Action**	Direct and indirect antioxidant; mitochondrial cofactor	Enhances transketolase activity; diverts glycolytic flux
**Level of Pathogenic Targeting**	Downstream: attenuates oxidative stress and its consequences	Upstream: reduces substrate supply for damage pathways
**Key Molecular Targets**	ROS (direct scavenger); regenerates glutathione, vitamins C and E; chelates metals; modulates NF-κB [25,26,27,28,29]	Transketolase (pentose phosphate pathway); indirectly reduces AGE formation, PKC activation, and hexosamine flux [8,36]
**Pharmacokinetic Properties**	Amphipathic (water and lipid soluble); good tissue penetration, including mitochondria [25]	Lipid-soluble pro-drug of thiamine; high bioavailability compared to thiamine salts [35,36]
**Effect on Glucose Metabolism**	May improve insulin sensitivity via AMPK activation [31]	Corrects intracellular thiamine deficiency; optimizes glucose utilization [8]

**Table 2:** Comparative clinical evidence profile of ALA and benfotiamine.

Feature	Alpha-Lipoic Acid (ALA)	Benfotiamine
**Volume of Clinical Evidence**	Large; multiple large-scale RCTs and a 4-year long-term study [4,5,6,7,32]	Limited; small, short-duration RCTs [10,11,39]
**Key Landmark Trials**	ALADIN I [4], ALADIN III [5], SYDNEY [6], SYDNEY 2 [7], NATHAN 1 [32]	BENDIP [11]; early combination studies [10,39]
**Consistency of Symptomatic Benefit**	Consistent across multiple trials; consistent improvement in TSS and other symptom scores	Suggestive but less consistent; some evidence for symptom improvement but less consistent
Some evidence from one long-term trial (NATHAN 1)	Moderate; NATHAN showed a marginal, borderline significant effect [32]	Insufficient; no long-term trials available
**Evidence for Structural Modification**	Low; no improvement in nerve conduction studies in NATHAN 1 [32]; none; no improvement in nerve conduction studies in NATHAN 1 [32]; IENFD not assessed; no evidence of nerve regeneration	None; structural endpoints not evaluated in published trials
**Safety and Tolerability**	Well-tolerated; mild GI side effects at higher doses [7]	Well-tolerated; favorable safety profile [11]

Note: These judgments are qualitative and based on the authors’ interpretation of the available literature, not on a formal evidence-grading system.

**Table 3:** Synthesis of comparative evidence and therapeutic positioning.

Criterion	Alpha-Lipoic Acid (ALA)	Benfotiamine
**Strength of Mechanistic Rationale**	Well-established; supported by extensive in vitro and in vivo data [25,26,27,28,29,30,31]	Well-established; strong biochemical and preclinical rationale [8,35,36,37,38]
**Strength of Clinical Evidence (Symptomatic)**	Consistent across multiple RCTs, including short-term and one long-term trial [4,5,6,7,32,33,34]	Limited; derived from short-duration (≤6 weeks), symptom-only studies [10,11,39]
**Strength of Clinical Evidence (Disease-Modifying)**	Insufficient; no demonstration of structural regeneration or consistent neurophysiological improvement [32]	Absent; no long-term or structural endpoint trials published
**Overall Level of Clinical Support**	More mature evidence base, but still inconclusive for disease modification	Promising based on mechanism, but clinically unconfirmed
**Current Therapeutic Positioning**	Adjunctive option for symptomatic relief in mild-to-moderate DPN; possible stabilization role	Not recommended for routine clinical use; may be considered only in documented thiamine deficiency or as part of an experimental nutritional strategy
**Key Evidence Gaps**	Effect on structural endpoints (IENFD, CCM); efficacy in advanced DPN	Long-term RCTs with structural and functional endpoints; identification of responder populations (e.g., based on thiamine status)

The labels and judgments in this table represent the authors’ qualitative interpretation of the available literature. No formal evidence-grading system (e.g., GRADE) or quantitative bias assessment was applied. Readers should interpret these summaries as descriptive, not as statistically derived certainty levels.

**Table 4:** Summary of major clinical trials of alpha-lipoic acid (ALA) in diabetic peripheral neuropathy.

Trial Name (Year)	Sample Size (*n*)	Diabetes Type	Intervention (Dose, Route, Duration)	Primary Endpoint	Main Results	Adverse Events	Risk of Bias/Limitations
ALADIN I (1995) [5]	328 (260 completers)	Type 2	IV ALA 1200 mg, 600 mg, 100 mg, or placebo daily for 3 weeks	Total Symptom Score (TSS)	TSS reduction: 600 mg: –5.0 (–63.5%, *p* < 0.001); 1200 mg: –4.5 (–58.6%, *p* = 0.003) vs. placebo	AEs: 32.6% (1200 mg), 18.2% (600 mg), 13.6% (100 mg), 20.7% (placebo)	Short duration (3 weeks); no long-term follow-up
ALADIN III (1999) [6]	509 (ITT: 509)	Type 2	IV 600 mg/day for 3 weeks, then oral 600 mg t.i.d. for 6 months vs. placebo	TSS; NIS	No significant difference in TSS at 7 months; NIS improvement after 19 days (*p* = 0.02) but not at 7 months (*p* = 0.09)	No difference in AE rates between groups	High intercenter variability; primary outcome negative
SYDNEY (2003) [7]	120	Type 1 and 2	IV ALA 600 mg vs. placebo, 5 days/week for 14 treatments (3 weeks)	Change in daily TSS	TSS improved by 5.7 points (ALA) vs. 1.8 points (placebo) (*p* < 0.001); all TSS items improved	Not reported in abstract; described as safe	Short duration; no long-term follow-up
SYDNEY 2 (2006) [8]	181 (ITT: 181)	Type 1 and 2	Oral ALA 600 mg, 1200 mg, 1800 mg, or placebo once daily for 5 weeks	Change from baseline in TSS	TSS reduction: 600 mg: −4.9 (51%); 1200 mg: −4.5 (48%); 1800 mg: −4.7 (52%) vs. placebo: −2.9 (32%) (all *p* < 0.05)	Dose-dependent nausea, vomiting, vertigo; 600 mg best risk–benefit	Short duration (5 weeks); no structural endpoints
NATHAN 1 (2011) [9]	460 (ITT: 460)	Type 1 and 2	Oral ALA 600 mg once daily vs. placebo for 4 years	Composite: NIS-LL + 7 neurophysiological tests			

**Table 5:** Summary of major clinical trials of benfotiamine in diabetic peripheral neuropathy.

Trial Name (Year)	Sample Size	Diabetes Type	Intervention	Duration	Primary Endpoint	Main Results	Limitations
Stracke et al. (1996) [11]	24	Type 1 and 2	Benfotiamine + B6/B12 vs. placebo	12 weeks	Nerve conduction velocity	Improved peroneal NCV (*p* = 0.006)	Very small sample; combination therapy
Winkler et al. (1999) [40]	36	Type 1 and 2	Various benfotiamine regimens	3 weeks	Symptom scores	Improvement in all groups (*p* < 0.01)	Small sample; very short duration; no placebo in all arms
BENDIP (2008) [12]	165 (ITT:133)	Type 1 and 2	Benfotiamine 600 mg, 300 mg, or placebo	6 weeks	NSS	NSS *p* = 0.033 (PP)/*p* = 0.055 (ITT); TSS not significant	Short duration; no structural endpoints; ITT borderline

## Data Availability

No new data were created or analyzed in this study. Data sharing is not applicable to this article.

## Abbreviations

The following abbreviations are used in this manuscript: DPN, diabetic peripheral neuropathy; ALA, alpha-lipoic acid; ROS, reactive oxygen species; AGE, advanced glycation end products; RAGE, receptor for advanced glycation end products; PKC, protein kinase C; RCT, randomized controlled trial; TSS, Total Symptom Score; NIS, Neuropathy Impairment Score; NIS-LL, Neuropathy Impairment Score–Lower Limbs; IENFD, intraepidermal nerve fiber density; CCM, corneal confocal microscopy; AMPK, AMP-activated protein kinase; GAPDH, glyceraldehyde-3-phosphate dehydrogenase; DCCT, Diabetes Control and Complications Trial; EDIC, Epidemiology of Diabetes Interventions and Complications; BENDIP, Benfotiamine in Diabetic Polyneuropathy; MeSH, Medical Subject Headings; PICO, Population, Intervention, Comparison, Outcome.
